# The Family Alliance Model: A Way to Study and Characterize Early Family Interactions

**DOI:** 10.3389/fpsyg.2017.01441

**Published:** 2017-08-23

**Authors:** Nicolas Favez, France Frascarolo, Hervé Tissot

**Affiliations:** ^1^Faculty of Psychology and Educational Sciences, University of Geneva Geneva, Switzerland; ^2^Department of Psychiatry, University Hospital Center and University of Lausanne Lausanne, Switzerland

**Keywords:** family alliance, Lausanne Trilogue Play, PicNic Game, triadic interactions, infant development, coparenting

## Abstract

The aim of this paper is to present the family alliance (FA) model, which is designed to conceptualize the relational dynamics in the early family. FA is defined as the coordination a family can reach when fulfilling a task, such as playing a game or having a meal. According to the model, being coordinated as a family depends on four interactive functions: participation (all members are included), organization (members assume differentiated roles), focalization (family shares a common theme of activity), affect sharing (there is empathy between members). The functions are operationalized through the spatiotemporal characteristics of non-verbal interactions: for example, distance between the partners, orientation of their bodies, congruence within body segments, signals of readiness to interact, joint attention, facial expressions. Several standardized observational situations have been designed to assess FA: The Lausanne Trilogue Play (with its different versions), in which mother, father, and baby interact in all possible configurations of a triad, and the PicNic Game for families with several children. Studies in samples of non-referred and referred families (for infant or parental psychopathology) have highlighted different types of FA: disorganized, conflicted, and cooperative. The type of FA in a given family is stable through the first years and is predictive of developmental outcomes in children, such as psychofunctional symptoms, understanding of complex emotions, and Theory of Mind development.

## Introduction

Family relations exert a unique influence on infant social and cognitive development (Feinberg, 2003; McHale, 2007; Favez et al., 2009). The concept of “family” has been used extensively in the developmental literature to encompass diverse social systems whose common denominator is to be “larger than dyads.” Several models of family functioning have been proposed, such as the Circumplex model (Olson et al., 1989), the family competence model (Beavers and Hampson, 1990), the McMaster model (Epstein et al., 2003), and the Security in the Interparental Subsystem model (Cummings and Davies, 2010). Most of these models are inspired by and in continuation of the structural approach of Minuchin (1974). These models highlight family-level dimensions such as cohesion (i.e., the emotional closeness between family members) or adaptability (i.e., the aptitude of the family system to respond adequately to internal and external demands); moreover, they allow one to conceptualize the interrelation between family dynamics and individual functioning. However, they were not specifically designed for the early family; an understanding of the relational dynamics of a system that includes an infant necessitates taking into account the particularities of the relations and interactions with a very young child (in particular, an emphasis on non-verbal behaviors, as the baby does not have language). The family alliance (FA) model has been conceptualized to this aim (Fivaz-Depeursinge and Corboz-Warnery, 1999). To understand the specificity of the model, one must be aware of its background: on the one hand in order to situate it in the continuation of studies that led to progressively enlarging the view of the significant relational context for the infant from the mother-infant dyad to multiperson systems (including the father and other close relatives such as siblings), and on the other hand in order to consider the interdependence between two levels of “relational reality,” that is, the individuals’ representations of the relational world and the behaviors implemented in the interactions with others (Stern, 1985; Reiss, 1989; Favez, 2010).

## Theoretical and Empirical Background of the Model

Historically, the emphasis of developmental studies was first put on the mother–infant relationship, the mother being in most cases the main caretaker in occidental societies. These studies have shown the interrelation between the mental state of the mother, her parenting behaviors, and the social and affective development of the child (Grolnick and Gurland, 2002). First, maternal representations of the relational world (in particular of herself as a mother, of the baby, and of herself in relation to her baby) influence the mother’s aptitude to be emotionally available, to understand the behaviors of her infant, and to respond appropriately to her infant’s needs (Benoit et al., 1997; Mayseless, 2006; Biringen and Easterbrooks, 2012). Maternal representations may be to a certain extent “distorted” as a consequence of her relational history, or depending on her personality or possible psychopathology; globally, distorted refers to a difficulty in the mother to form a representation of the baby as autonomous and different from her (Slade et al., 2005). Second, studies have shown that daily mother–infant interactions are highly patterned: visual and affective exchanges begin, for example, with a “salutation” phase, followed by a “dialog” phase with several bouts of engagement and pause, and end with a “termination” phase. Within this structure, mutual behaviors of the mother and the infant are contingent on one another (i.e., each behavior of a partner is an answer to the behavior of the other, and behaviors are produced within a proper time frame); moreover, the mother continuously adjusts her stimulations of the child to the child’s affective state. Finally, the interactions are organized around a theme such as a peek-a-boo game (Brazelton, 1974; Stern, 1977). Distorted representations may induce alterations in the structure of the interaction: a mother with depression tends, for example, to have a representation of the baby as not being interested in interacting with her; she may then enact overstimulating behaviors in order to force the interaction (i.e., by not respecting the necessary pauses in the interaction that the baby needs to take in order to avoid excessive arousal), or, in contrast, she may show understimulating behaviors, as she lacks the motivation to interact. As a consequence, the baby may withdraw quickly from the interaction by not looking at the mother, which will be understood by her as confirmation of the lack of interest of the baby. This dysfunctional pattern thus tends to repeat itself, which has an impact on the development of the infant’s emotional regulation skills: the infant may progressively generalize withdrawal to all social situations (Anders, 1989; Tissot et al., 2011). Disturbances in daily relationships were identified as the main mediating variable by which emotional difficulties or a psychopathology affecting the mother have an impact on the child (Goodman and Gotlib, 1999).

The father–child relationship has been studied in a second step, as the father was long considered to exert only a distal influence on the development of the infant (Frascarolo and Favez, 1999). However, the important social changes that Western societies went through at the end of the 1960s and the beginning of the 1970s (with, for example, the removal of “paternal power” from civil codes in countries such as France in 1970 or Switzerland in 1977) gave rise to a generation of “new fathers,” who began to claim responsibilities in the care of their children, even in the child’s early years. Following these changes, numerous studies have been dedicated to the development of the father–child relationship, replicating the protocols that have been used in studies on mother–child relationships. The results have shown similarities between mothers and fathers in the aptitude to understand the emotional signals of their babies (for example, both parents are equally sensitive to their child’s needs, and they are both able to adjust their stimulations to the affective state of the child); moreover, father–infant interactions are patterned similarly to mother–infant interactions, but with differences in the preferred themes of interactions: fathers are more prone to use physical play and to be unpredictable in their stimulations of the child (see Parke, 1996, and Lamb, 2010, for comprehensive reviews). Finally, father psychopathology also has a negative impact on the development of the child (Connell and Goodman, 2002; Ramchandani et al., 2008).

The results of these studies have thus shown that both parents are significant partners for the child, which brought to the fore the question of the coordination between mother and father concerning their respective beliefs and expectations about parenting, as well as their daily interactions with the infant. In this regard, empirical evidence has shown a true emergent effect when the family interacts as a triad. Parke and O’Leary (1976) have, for example, observed that the interactions between one parent and the infant are different when they happen in the presence or absence of the other parent; these researchers coined the term “second-order effect” to describe the influence of one relation on another: for example, both mothers and fathers show less negative emotion and are less involved in the interaction when they interact with the child in the presence of the other parent than when they are alone with the child (Johnson, 2001; Udry-Jørgensen et al., 2016). It thus became obvious that a comprehensive assessment of the social context of the development of the child could not be done by only taking into account each dyad separately. Stated differently, the dyadic systems (mother-infant and father-infant) cannot be added to one another; the triadic level – the way that interactions are organized when mother, father, and infant are together – is a level in its own right, offering a particular context in which the child lives a unique experience.

The acknowledgment of the importance of the triadic level also occurred as a result of studies that have shown the impact of the relationship between the parents on the child. Studies on interparental conflict have shown the psychological and behavioral negative consequences for school-aged children who are raised in a conflicted home (Cummings and Davies, 2010). Similarly, studies on the consequences of divorce have shown that an enduring post-divorce interparental conflict is the main variable that explains children’s difficulties in coping with the separation (Amato and Keith, 1991; Amato, 2001); in contrast, cooperation between the ex-spouses tends to temper the negative effects of divorce (Amato and Rezac, 1994; Amato et al., 1995; Hetherington and Stanley-Hagan, 2002; Adamsons and Pasley, 2006). In infancy, interparental conflict also affects children. First, there is a direct effect: repeatedly witnessing intense and unresolved conflicts between the parents has consequences on the emotional regulation skills of the infant. Atypical vagal tone has thus been observed that tends to be generalized to any stressful situation; the child may then have a tendency to social withdrawal when facing novelty (Crockenberg et al., 2007; Lindblom et al., 2014). Second, there is an indirect effect: parent–infant relationships are in themselves affected by the disturbances in the parent–parent relationship. This phenomenon was described as the “spillover effect” (Fainsilber Katz and Gottman, 1996; Crockenberg and Leerkes, 2003a): parents engaged in a conflict show more anger and irritation in their interactions with the infant, have less empathy and emotional availability, and display controlling and rejecting behaviors – all in all, behaviors that are similar to those described in parents with a psychopathology (Webster-Stratton and Hammond, 1999; Davies et al., 2002). The representations the parents have of the infant are also modified – or distorted; for example, parents describe the child as being more difficult than external informants do (Fainsilber Katz and Gottman, 1991, 1996).

The development of the child is thus affected by the cooperation and the conflict between the parents. This cooperation, or lack of it, has been conceptualized as the coparenting relationship – that is, the support parents bring to each other, or not, in their relation with the child at instrumental and emotional levels (McHale and Lindahl, 2011; Beaton et al., 2013, for a review; Minuchin, 1974). Cooperative coparenting comprises support and warmth between the parents; disagreements are resolved by negotiation (McHale, 1997). On the other hand, when there is an underlying and enduring conflict, parents may show hostility to each other: each parent may try to undermine what the other has done, or be critical and disparaging of the parenting work of the other. Another possible issue of conflict is skewed coparenting: one of the parents withdraws from family life; thus, there is no overt conflict, but no real coparental relationship either. Finally, the conflict may have as a consequence a lack of positive affects and emotional support, even though the two parents are still cooperating at an instrumental level (McHale et al., 2002). All of these variations in coparenting are non-optimal contexts for the development of the child; if they become chronic, they are linked with adaptive problems and even psychopathology in the child (McHale and Rasmussen, 1998; McHale and Fivaz-Depeursinge, 1999; Schoppe et al., 2001; Schoppe-Sullivan et al., 2009; LeRoy et al., 2012; see the meta-analysis by Teubert and Pinquart, 2010).

It is of note that the emphasis has been put on each parent and on their collaboration; the child should not, however, be considered merely a passive partner in the interactions, even though her/his contribution has often been overlooked (McHale and Crouter, 2003). First, studies have shown that the infant has very early (at as early as 3- to 6-months-old) the attentional and behavioral aptitudes to interact at a triangular level: on the one hand in person-person-object situation (PPO), by sharing attention toward an object with the adult or by sharing intention for action (Trevarthen and Hubley, 1978; Butterworth, 1998; Rochat and Striano, 1999; Striano et al., 2007; Moreno-Núñez et al., 2015; Reddy, 2015), and on the other hand in person-person-person situations (PPP), for example by visually following the exchange between the parents (Fivaz-Depeursinge et al., 2005, 2007), or by being engaged in the interaction with two peers at a time (Selby and Bradley, 2003). Interestingly, studies which have compared the two types of situations have shown that the PPP situations elicit a greater behavioral arousal in the infant (for example, more smiles or hand movements) than the PPO situations (Tremblay and Rovira, 2007). These triangular aptitudes are used by parents when they compete for the attention of the child in order to distract themselves from an ongoing conflict (Fivaz-Depeursinge and Favez, 2006), a process that may be the origin of the triangulation process described by Minuchin (1974) in families with older children, where children are used as a scapegoat or go-between to regulate the tensions between the parents. Second, the temperament of the child (in terms of disposition to regulate emotions) has an effect on the parenting behaviors of the parents and also on the interparental relationship. Parents of a so-called difficult child (slow to warm up, irritable, difficult to calm or fussy) develop a style of parenting that is all the more responsive during the first months, but in the end, their unsuccessful efforts to calm the child can lead them to show a parenting style that is colder and more distant (Crockenberg and Smith, 1982; van den Boom and Hoeksma, 1994; Sanson and Rothbart, 1995). Similarly, the probability of interparental conflict is higher (Stifter, 2003), especially when there is preexisting dissatisfaction in the marital relationship (Crockenberg and Leerkes, 2003b; McHale et al., 2004; Schoppe-Sullivan et al., 2007). In our own study on the transition to parenthood, we have seen that the child’s temperament at 3 months and the coparental interactions during the pregnancy are the two best predictors of the quality of the triadic interactions at 18 months (Favez et al., 2013).

## The FA Model

Several findings of the studies we have reviewed may be highlighted, as they constitute building blocks for the FA model. First, studies have shown that every partner brings a significant contribution to the family interaction, including the infant. Moreover, even when one parent is not active in the interaction, the parent’s mere presence is sufficient to introduce modifications into the interaction between the other parent and the child. This points to the importance of considering the inclusion of each and every partner of the family in the interaction. Second, a successful interaction implies organization of the behaviors of the partners: there should be contingency between the behaviors, respect of turn taking, and no competitive behaviors – especially in the stimulations of the parents toward the child. Third, interactions have a theme: even at a non-verbal stage, a play has a narrative plot around which the behaviors are articulated. Finally, the affective or emotional tone of the exchanges are of paramount importance; studies have shown, for example, that coparental coordination that is only instrumental and devoid of affects does not constitute a functional coparenting relationship.

Based on these findings, the FA model has been designed to assess the degree of family engagement and coordination in any joint activities (Fivaz-Depeursinge and Corboz-Warnery, 1999). It allows one to describe relational dimensions specific to “beyond-the-dyad” systems and simultaneously takes into account all the members of the family who are taking part in an interaction. Besides the studies mentioned earlier, the model is inspired by the symbolic interactionism approach (Blumer, 1969) and by the ecosystemic model (Keeney, 1979), which put non-verbal communication and contextual information at the core of human interactions. According to the model, FA depends on four interactive functions: participation, organization, focalization, and affect sharing. These functions are hierarchically embedded; each is a necessary condition for the achievement of the next.

- Participation: The most basic function is that when a family is interacting, all family members should be included; that is, they are all available to interact and are interested in each other.
- Organization: When all the family members are included in the interaction, the family has to organize turn taking and/or to attribute differentiated roles according to the aim of the interaction (there might be one narrator, for example). Each family member has a role to play in family interactions.
- Focalization: When all the partners are included and their roles distributed and respected, the co-construction of an activity is possible; each person’s attention or gestures have to be focused on the same theme in order to co-construct an activity.
- Affect sharing: Finally, when there is joint attention between partners, emotional attunement is possible; affects circulate between them and there is mutual empathy.

The functions are implemented in the interaction according to the spatial and temporal features of the partners’ behaviors. The distance between the partners and the orientation of their bodies form a transactional space that allows emotional exchanges (Hall, 1966; Argyle, 1972; Kendon, 1977). Moreover, the positions of the different body parts inform about the partner’s readiness to interact and determine the partner’s level of engagement in the interaction. Communication is clear and engagement is high when all the body parts are congruent (face, torso, and pelvis are all oriented toward the partner); incongruence between body parts results in ambiguity about the engagement (e.g., the face is oriented toward the partner, while the torso and pelvis are oriented away from the partner; Scheflen, 1973). Participation thus implies that all partners signal their readiness to interact by their body positions and orientation, especially the most basic level of the pelvises: a geometric figure could be drawn between the center points of each partner’s pelvis; the torsos are oriented toward this transactional space. Organization is most often indicated by the inclination of the torsos (e.g., the speaker leans forward while the listener is seated straight or leaning back on the chair; each change in roles is marked by a change in body position), by the gestures in congruence with the role (e.g., the speaker uses specific hand gestures), and by the contingency in the behaviors of the partners (e.g., the speakers hold the floor each in turn and not at the same time). Focalization is determined by joint visual attention (e.g., when all partners look at the same object, or when all listeners look at the speaker) and by the development of a common theme (e.g., a consistent topic through the verbal or non-verbal exchanges). Finally, affect sharing is determined by facial expressions, verbal exchanges, and vocal tone (e.g., the emotional expressions of all partners are attuned).

## The Situations of Observation

In order to allow the assessment of FA through systematic observation of interactions, we have developed several observational situations for the laboratory. The two main situations are the Lausanne Trilogue Play (LTP), specifically designed for triadic interactions and aimed at assessing how the family interacts in several predetermined configurations, and the PicNic Game (PNG), specifically designed for families with several children and aimed at assessing how the family organizes itself spontaneously. These situations are windows into family functioning; their basic premise is the fact that being able to resolve a small task (to follow the instructions and to cope with the stress of being observed) in a laboratory setting is representative of the aptitude of the family to solve more important tasks (Beavers and Hampson, 1990; Kerig, 2001).

### The LTP: Assessment of Triadic Interactions

The aim of the LTP for the parents and the infant is to play together and thus to have fun and share a pleasurable moment. Its scenario is structured in four parts, which aim to make the triad go through all possible configurations of daily triadic interactions (Corboz-Warnery et al., 1993):

(1) One parent plays with the infant while the other parent is in a third-party position, as participant-observer.
(2) The parents switch roles.
(3) The three partners play together.
(4) The parents have a discussion with each other, while the infant is the third party.

**FIGURE 1:**
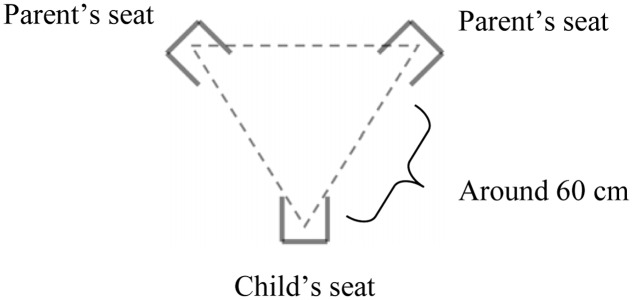
Triangular arrangement of partners in the Lausanne Trilogue Play.

The parents sit on chairs arranged in a triangle at a distance that is favorable to the establishment of a dialog (according to the so-called “F-formation” that designates the optimal interaction space, where each interaction partner has equal and direct access to the space established by the group of partners as a whole; Kendon, 1977). These chairs cannot be moved. The infant is in a baby-reclining chair that can be oriented toward one parent, toward the other, and between the two (**Figure 1**). The following instructions are given: “*We ask you to play together as a family. We will ask you to play a scenario with four different parts. In the first part, one of you plays with the child, the other one being simply present. In the second part, roles are reversed. In the third part, you both play with the child together. In the last part, you will talk together and you will leave the child on his own for a little while*.” The parents can decide how long each part of the scenario is to last; empirically, it was found that the average duration during the first year is around 12 min for the entire game (Favez et al., 2006b). The instructor mention to the parents this usual duration as an indication. Parents are also asked to signal either verbally or with a hand gesture when they decide that the game is finished. The design is adapted to the age; thus, after 12 months, the parents and child sit around a small round table and some toys are at hand (spoons, socks, and plush toys). The entire play is videotaped.

Variations of the LTP have been developed for specific assessments (see **Table 1** for details). The prenatal LTP allows us to assess how expecting parents can anticipate and enact interactions with their baby-to-come, simulated by a doll; the scenario is the same as for the standard LTP, accompanied by an appropriate procedure in order to prepare the parents for this unusual pretend-to-be game (see Carneiro et al., 2006 for the details). The still-face LTP allows the assessment of the extent to which the baby can resort to the second parent when one parent poses a still-face: in the first part, mother, father, and baby interact together; in the second part, one of the parents interacts with the baby while the other is simply present; in the third part, the active parent posits a still-face while the second parent stays in the role of being simply present; finally, in the fourth part, all three play again together (Fivaz-Depeursinge et al., 2005). The LTP standard, the prenatal LTP and the still-face LTP may be used with primiparous families as well as with multiparous families. The Lausanne Family Play (LFP) has been designed for families with several children; it follows the classic LTP scenario (in the first two parts, the active parent plays with all children, and in part three, the family plays together). Several toys are at hand: a “family” of lions, of ducks, and of toy cell phones (as many as there are members of the family). These specific toys have been selected as they allow to play in group.

**Table 1:** Summary of observational situations.

Situation	Structure	Setting
Standard LTP	Four parts:	When the child is <12 months: The parents sit on chairs arranged in a triangle. These chairs cannot be moved. The infant is in a baby-reclining chair that can be oriented toward one parent, toward the other, and between the two.
	(1) One parent plays with the child, while the other is simply present.	When the child is >12 months: parents and child are seated at a round table.
	(2) The parents switch roles.	Several toys are at hand (wooden blocks, animals).
	(3) Both parents and the child play together.	
	(4) The parents discuss and the child is simply present.	
Prenatal LTP	Same as standard LTP.	The “baby” is a doll presented in a basket. The basket and the parents are arranged in a triangle. The doll has the weight of a newborn baby; it has no clear face (i.e., no eyes, no mouth).
Still-face LTP	Four parts:	Same as standard LTP.
	(1) Both parents and the child play together.	
	(2) One parent plays with the child, while the other is simply present.	
	(3) The parent who played with the child posits a still-face. The other parent is simply present.	
	(4) Both parents and the child play together.	
LFP	Same as standard LTP. In the first two parts, the active parent plays with all children, and in part three, the family plays together.	Parents and children are seated at a round table. Several toys are at hand (lions, ducks, and cell phones – as many as there are participants).
PicNic Game	Parents are asked to organize the picnic from the preparation of the meal to the end, including tidying up.	A green carpet (4 m × 4 m) is used to delimit the space at disposal for the game. There is also a bench (similar to those in public parks), a table, chairs (as many as there are participants), a dinnerware set stored in a basket, and several age-appropriate toys in a bag (e.g., cars, dragons, knights, and cubes).


*LTP, Lausanne Trilogue Play; LFP, Lausanne Family Play.*

The LTP situation restrains the interactions and the family members have to adapt their communication, verbal but mostly non-verbal, to the situation’s requirements. It is first and foremost through bodily configurations that each family will be able to implement an interaction context that both respects the instructions and allows an emotionally satisfying exchange between the partners. Thus, if one takes, for example, the first part of the LTP, the parent who has to stay in the background faces a delicate situation: through the positioning of the chairs, this parent finds her/himself sitting next to the other parent and at the same distance from the baby, but at the same time, has to signal that she/he is not available, which she/he often does by holding her/himself with her/his chest back, leaning against the back of the chair. The active parent generally leans forward to interact with the baby. The change from one part to another allows us to test the way the family negotiates transitions and reorganizes itself after a change of configuration.

Variations in the accomplishment of the functions yield several types of alliances. An alliance may be functional whereby the family succeeds in setting up a context allowing triadic exchanges, or dysfunctional when such a context cannot be set up or only at the expense of negative emotions. There are two types of dysfunctional alliances:

(i) When participation is not fulfilled, the systematic (self-)exclusion of one of the family members leads to a “disorganized alliance.” A parent may exclude her/himself from the family interactions either by turning away from the family or by being systematically emotionally absent – stonewalling is one example, or a dyad may leave no room for the third partner: a mother’s extreme gatekeeping with the child may systematically leave the father aside. Finally, both parents may exclude the baby by not placing her/him in the optimal conditions to interact with them (by seeking out the baby’s attention at inadequate moments, or by not settling the baby in an appropriate position or by overstimulating her/him, which will cause the baby to withdraw, for example).
(ii) In some families, even though all members are included in the interaction, interferences or competition appears; the parents then show difficulties in distributing roles, negotiating activities together, and cooperating. Organization is thus not fulfilled. Competing and uncoordinated activities give the impression that each parent tries to attract the child’s attention and enter into a privileged relationship with the child to the detriment of the exchange between the other parent and the child. These interactions reveal a “conflictual alliance.”

After the participation and organization are fulfilled, the basic systemic prerequisites are also fulfilled and a line can be drawn between functional and dysfunctional alliances. The functional alliance is then assessed as “cooperative”; two subtypes of cooperative alliance are observable:

(iii) Even though all members are included in the interaction and roles are distributed, achieving joint activities may be a struggle; the co-construction may go through a certain number of false starts and the emotions may be a bit forced. The lack of creativity produces games that are quite flat, accompanied by a neutral affective atmosphere, even though empathy is present between the family members. Affect sharing is thus not completely fulfilled. The small mistakes, inevitable in any communication, are solved more slowly and provoke a tense atmosphere, the task developing in a fitful way. The transitions between different relational configurations may confuse and break the task’s flow. The main characteristics of this type of alliance are the efforts put in by everyone to succeed in the task and the lack of fluidity that ensues; this alliance is “cooperative stressed.”
(iv) Joint activities may be fluid and affects mainly positive; moreover, affects are shared and participants are emotionally attuned. Possible negative emotions in the baby are also acknowledged and regulated in the interaction. The alliance is then “harmonious cooperative.”

To summarize, the accomplishment of the four functions resulting in an harmonious cooperative alliance brings about an interaction with the following characteristics in the LTP: each partner’s participation in the game’s elaboration is according to the scenario; the respect of the successive roles required by the instructions occurs with a fluid transition between the different configurations that does not interrupt the flow of exchanges: the family members stay involved in the activities and enrich them as they go along; and the activity carried out is sustained, coherent, and consistent, with a narrative outline. The global affective atmosphere is warm and empathic. The partners are in contact with one another. This can be observed from their facial and vocal expressions, which reflect the affects shared by the different family members. If the child is not collaborating much or expresses negative emotions, the parents support the child in an adequate way and allow the child to regulate her/himself. Generally, positive affects are shared, observed in mutual smiles and light marks of humor.

### PNG: The Assessment of Interactions in Families with Several Children

The LTP has been designed mainly to assess triadic family interaction. Yet, clinical evidence and empirical studies have provided hints of the fact that parents may behave differently with their different children (Stern, 1971; Volling and Elins, 1998; McHale et al., 2000; Rauer and Volling, 2007), indicating that assessing family interactions with one child may not be sufficient for a comprehensive assessment of the interactive dynamics in families with multiple children. Observation of a system with more than three partners is a methodological challenge, however, given the complexity of multiple mutual exchanges that happen when several people interact together. The LFP presented earlier was a first attempt to take this challenge; however, to reduce complexity and still have predetermined interactive configurations, we asked parents to play with all their children simultaneously in the different parts of the LFP (having each parent interacting with each child separately would extend the number of parts and the length of the game beyond reasonable limits). The LFP thus does not allow us to assess whether subsystems are privileged by the family: for example, a closer interaction between father and son than between father and daughter. To realize such an assessment, a more open observational strategy is indicated, giving access to the way the family with multiple children organizes itself spontaneously when interacting as a group.

In family psychology, observation of real meals in naturalistic settings is one of the methodologies that has been used to gain access to spontaneous family group-level interactions (see, for example, McHale, 2004; Fiese et al., 2002, 2006; Spagnola and Fiese, 2007; Kuersten-Hogan and McHale, 2013). As a daily family activity, having a meal is a strongly patterned situation that is ideal for observing a family interacting according to its usual interaction schemas. Nonetheless, the variability of naturalistic meal contexts (e.g., available space, length of meals) makes comparisons between families difficult; moreover, the downside of daily routines is that they may not create sufficient activation of the family system to observe how the family faces novelty. The PNG has been designed for systematic observation (Frascarolo and Favez, 2005; Favez et al., 2016); its central theme is to pretend that a family is sharing a meal during a picnic.

**FIGURE 2:**
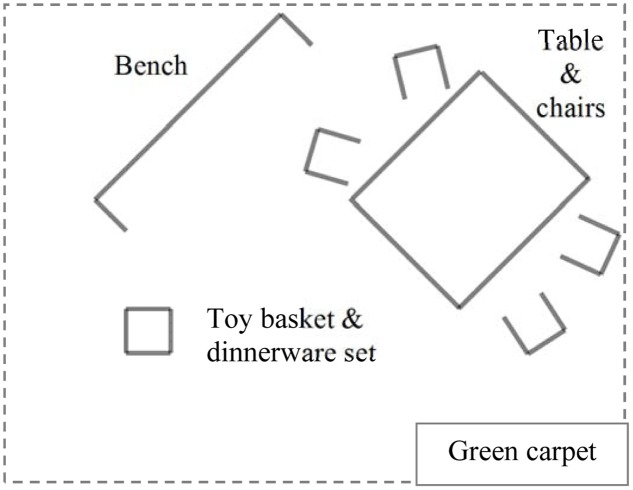
Arrangement of the PicNic Game.

To perform the PNG, a green carpet (4 m × 4 m) is used to delimit the space at the family’s disposal (**Figure 2**). There is also a bench (similar to those in public parks), a table, chairs (as many as there are participants), a dinnerware set stored in a basket, and several age-appropriate toys in a bag (e.g., cars, dragons, knights, cubes). Sufficient free space on the carpet is left so that the family can play seated on the ground. The aim is not for the family to demonstrate a real meal, but rather to perform a pretend play; the game allows researchers to have access not only to patterned daily rituals of the family (e.g., setting the table, tidying up at the end), but also to the family’s potential for creativity.

The instructions given to the family by the facilitator are as follows:

We ask you to pretend that you are having a picnic. Imagine you are going to a park with your child(ren) X (and Y). The green carpet represents the area of grass you can use; the edges of the carpet are limits not to be crossed. You have the picnic together on the grass, or on the bench, or at the table. If you picnic at the table, you can place the chairs as you wish. Here, in this basket, there is a dinnerware set. And in this/these bag(s), one per each child, there are toys. You organize everything as you wish. You are invited to tidy up when you are finished. Do take your time. In general, the whole game lasts about a quarter of an hour. You can begin when I leave. I will be behind the window. Please call me when you are finished or if you have any trouble.

The game is video recorded for coding purposes with a wide-angle camera. The facilitator stands in an adjacent room, behind a window. When the parents signal that the game is finished, the facilitator goes back into the laboratory and gives the family a quick debriefing about the stress they may have felt during the situation. We also ask parents if they feel that their game in the LTP was representative of their everyday interactions (Favez et al., 2017). In clinical settings, the video recording can be used for a video-feedback procedure.

The accomplishment of the four functions of the FA model brings about an interaction with the following characteristics in the PNG: each partner participates in the game; even the youngest infants are included. The game is structured and roles are distributed; different parts are distinguishable (e.g., preparing the meal, dressing the table, eating, playing, and cleaning) and the game occurs as a narrative plot. All partners are focalized on the theme of the activity, and the game has a flavor of genuineness or of fun, with authentic affects. The two parents work together as a coparental team by supporting each other in their parental tasks (setting limits for the children, deciding who will look after which child, who will “cook” and who will dress the table, for example). There might even be more “conjugal” moments, if the children begin to play together (signs of affection between the parents, talking about a conjugal theme, for example). The global affective atmosphere is warm and empathic. The partners are in contact with one another. This can be observed from their facial and vocal expressions, which reflect the affects shared by the different family members. If the children do not want to collaborate, the parents set clear limits to allow the game to happen.

To date, our data allowed us to determine whether the alliance is more or less functional in the PNG according to a dimensional perspective; we do not yet have a validated categorical perspective with different types of alliances, as we do in the LTP.

## The Stability of FA and Its Impact on the Development of the Child

The systematic assessment of the alliance is carried out with the help of validated assessment tools, such as the Family Alliance Assessment Scales (Favez et al., 2011) in the LTP and the Revised-PicNic Assessment Scales (Favez et al., 2016) in the PNG – both of these instruments include dimensions specific to their respective observational situations. This has allowed us to highlight, in the context of longitudinal studies with non-referred families, that the FA is stable in time, from the fifth month of pregnancy (alliance measured in the prenatal LTP) to the end of the child’s second year and until the firstborn child has reached the age of 5 years (alliance measured in the LFP and in the PNG). For example, coparenting dysfunctional processes such as competitive behaviors may be observed at every age in a given family: when the parents-to-be play with a doll during pregnancy, instances of competition may be shown when parents argue angrily about whom the doll is supposed to resemble the most; at 3 and 9 months, the parents might offer divergent stimulations to the baby in order to capture her/his attention; at 18 months and later, the competition may be verbal with mutual contradictions, contradictory instructions given to the child, and angry comments by the parents addressed to one another (Favez et al., 2006a, 2012; Frascarolo et al., 2016).

This stability suggests that triadic (and polyadic) interactions are patterned, just as dyadic interactions are. This confirms that the observation of interactive behaviors is a sound methodology to assess early family relationships; in addition, it allows us to include infants and young children for whom other assessment strategies are not relevant. Because they are patterned, interactions observed in the laboratory may be considered meaningful and to reflect real-life interactions of the family. From a developmental point of view, the stability of interactions implies that FA constitutes a stable relational context in which the child learns social skills; this relates to the concept of “practicing family” proposed by Reiss (1989), namely, the transmission of values and ways of behaving through the usual interactive patterns of the family. These contexts may be more or less favorable to the cognitive and emotional development of the child: data from our longitudinal studies show, for example, that children from families with a dysfunctional alliance are less efficient in theory of mind tasks and in the understanding of complex emotions at age 5 years. Interestingly, these families are also the families in which parents report less social support from their environment (Favez et al., 2006a,b, 2012).

Dysfunctional alliances are predominant in families referred for parental psychopathology (e.g., postpartum depression), and alliance assessment has a good discriminant value between referred and non-referred families (McHale and Fivaz-Depeursinge, 1999; Fivaz-Depeursinge et al., 2000). The hierarchical structure of the model has been empirically validated by observation of interactions in non-referred families (Frascarolo et al., 2004): when a function of higher order is fulfilled (e.g., focalization), the functions of lower orders are always fulfilled (participation and organization), but the reverse is not true (e.g., participation does not imply organization, focalization, or affect sharing). Clinical practice has shown that the more severe the psychopathology in parents (e.g., mothers with a diagnosis of schizophrenic disorder, or with a major depression), the more difficult it is for the family to fulfill even the lower order functions. Accordingly, interventions may target the dimensions, beginning with participation in families in which including everyone is difficult, and then organization if participation is fulfilled but attributing roles generates conflicts, and so on (Favez et al., 2009). Interventions may be performed by using the situations of observation in several ways: first by direct intervention during the situations themselves, based on the family’s interactive behaviors and by suggesting alternative and non-problematic patterns, or by using the information stemming from the situation to prescribe rituals to be carried out at home between two sessions. Second, the films allow the use of video feedback, which is a powerful way to enhance family experience of relationships by supporting awareness of positive and negative interactive patterns (McDonough, 1993; Fivaz-Depeursinge et al., 2004).

## Conclusion

Developmental research findings have shown the importance of the family level for a comprehensive understanding of the child’s development. FA is a model designed to conceptualize family interactions in the early family, including one (or more) infants or young children; based on observable interactions, the qualification of family relationships in terms of alliances according to four interactive functions is an effective and valid way of making a diagnosis of family functioning. Two main situations of observation have been designed according to this model: the first one, the LTP (with its variations), has been used for research (e.g., McHale et al., 2004; Favez et al., 2006a; Hedenbro et al., 2006; Feldman, 2007; Von Klitzing et al., 1999a,b), as well as for clinical purposes (Fivaz-Depeursinge et al., 2000; Harrison, 2005) from the beginning of the 1990s. The second one, the PNG, has been used in research settings (Favez et al., 2012) and has begun to be used in clinical settings (Frascarolo and Favez, 2009; Bullens and D’Amore, 2013). These situations were mainly used in laboratory settings, but they may be also used at home, with some adjustments; this is especially true for the PNG as it has a less structured format than the LTP’s. The data from studies using these situations of observation show that the FA model is efficient to describe and assess family dynamics according to specific family-level dimensions – that is, dimensions that would be impossible to account for in assessing only individuals or dyads. Additional studies are now needed to assess the extent to which the FA model and its tools may be generalized across cultures – and specifically to non-Western ones. First, the situations of observation and their respective instructions may not be relevant to elicit patterned family interactions in any culture. Second, the behavioral indicators of a functional alliance may vary from one culture to another, similar to the cultural variations that have been observed in mother–infant interactions (e.g., Posada et al., 2002; Kärtner et al., 2010). Finally, the use of those situations of observation and their coding systems require a training, whether for clinical or research purposes. Workshops are organized on a regular basis by the Center for Family Studies in Lausanne (CEF); information can be obtained by contacting the authors of this article.

## Ethics Statement

The studies mentioned in this paper were approved by the Ethical Committee of the State of Vaud, Switzerland.

## Author Contributions

NF: Wrote the main parts of the manuscript. FF: Reviewed the manuscript and wrote specific parts regarding the PNG. HT: Reviewed the manuscript and wrote specific parts regarding the LTP.

## Conflict of Interest Statement

The authors declare that the research was conducted in the absence of any commercial or financial relationships that could be construed as a potential conflict of interest.
